# 1-[2,6-Dichloro-4-(trifluoro­meth­yl)phen­yl]-3,4-dimethyl­pyrano[2,3-*c*]pyrazol-6(1*H*)-one

**DOI:** 10.1107/S1600536812029789

**Published:** 2012-07-07

**Authors:** Hoong-Kun Fun, Wan-Sin Loh, B. K. Sarojini, B. J. Mohan, B. Narayana

**Affiliations:** a X-ray Crystallography Unit, School of Physics, Universiti Sains Malaysia, 11800 USM, Penang, Malaysia; b Department of Chemistry, P.A. College of Engineering, Mangalore 574 153, India; c Department of Chemistry, Mangalore University, Mangalagangotri 574 199, Mangalore, India

## Abstract

In the title compound, C_15_H_9_Cl_2_F_3_N_2_O_2_, the 1,6-dihydro­pyrano[2,3-*c*]pyrazole ring system is almost planar, with a maximum deviation of 0.0226 (14) Å, and forms a dihedral angle of 69.90 (6)° with the benzene ring. In the crystal, mol­ecules are linked into a helical chain along the *c* axis by C—H⋯O hydrogen bonds.

## Related literature

For background to and the biological activity of pyrazolone derivatives, see: Kokura *et al.* (2005 ▶); Sarojini *et al.* (2010 ▶); Vaid *et al.* (1986 ▶). For related structures, see: Ramsay & Steel (1985 ▶); Ahmad *et al.* (2011 ▶). For bond-length data, see: Allen *et al.* (1987 ▶). For the stability of the temperature controller used in the data collection, see: Cosier & Glazer (1986 ▶).
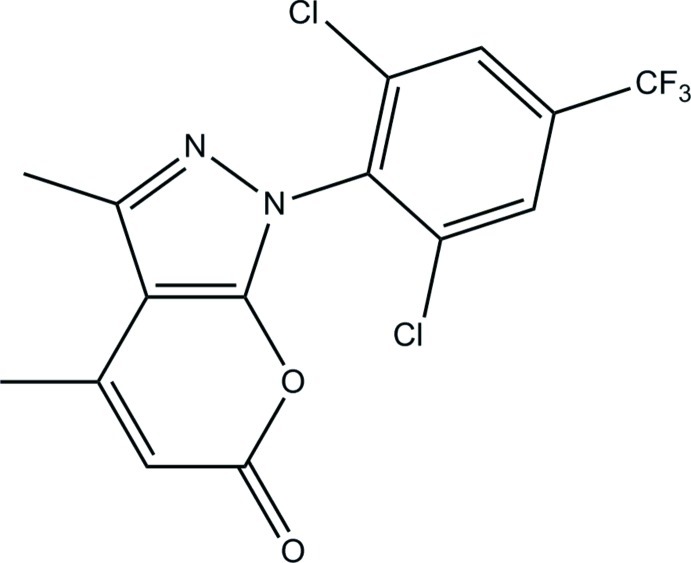


## Experimental

### 

#### Crystal data

C_15_H_9_Cl_2_F_3_N_2_O_2_
*M* *_r_* = 377.14
Orthorhombic, 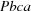

*a* = 13.3348 (2) Å
*b* = 14.2045 (2) Å
*c* = 15.9132 (3) Å
*V* = 3014.19 (8) Å^3^
*Z* = 8
Mo *K*α radiation
μ = 0.48 mm^−1^
*T* = 100 K
0.44 × 0.31 × 0.26 mm

#### Data collection

Bruker SMART APEXII CCD area-detector diffractometer
Absorption correction: multi-scan (*SADABS*; Bruker, 2009 ▶) *T*_min_ = 0.819, *T*_max_ = 0.888
24894 measured reflections
4382 independent reflections
3706 reflections with *I* > 2σ(*I*)
*R*_int_ = 0.030

#### Refinement

*R*[*F* ^2^ > 2σ(*F* ^2^)] = 0.036
*wR*(*F* ^2^) = 0.096
*S* = 1.03
4382 reflections
219 parameters
H-atom parameters constrained
Δρ_max_ = 0.43 e Å^−3^
Δρ_min_ = −0.52 e Å^−3^

### 

Data collection: *APEX2* (Bruker, 2009 ▶); cell refinement: *SAINT* (Bruker, 2009 ▶); data reduction: *SAINT*; program(s) used to solve structure: *SHELXTL* (Sheldrick, 2008 ▶); program(s) used to refine structure: *SHELXTL*; molecular graphics: *SHELXTL*; software used to prepare material for publication: *SHELXTL* and *PLATON* (Spek, 2009 ▶).

## Supplementary Material

Crystal structure: contains datablock(s) global, I. DOI: 10.1107/S1600536812029789/is5160sup1.cif

Structure factors: contains datablock(s) I. DOI: 10.1107/S1600536812029789/is5160Isup2.hkl

Supplementary material file. DOI: 10.1107/S1600536812029789/is5160Isup3.cml

Additional supplementary materials:  crystallographic information; 3D view; checkCIF report

## Figures and Tables

**Table 1:** Hydrogen-bond geometry (Å, °)

*D*—H⋯*A*	*D*—H	H⋯*A*	*D*⋯*A*	*D*—H⋯*A*
C11—H11*A*⋯O2^i^	0.95	2.44	3.3405 (18)	157

Symmetry code: (i) 
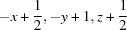
.

**Fig. 1.**
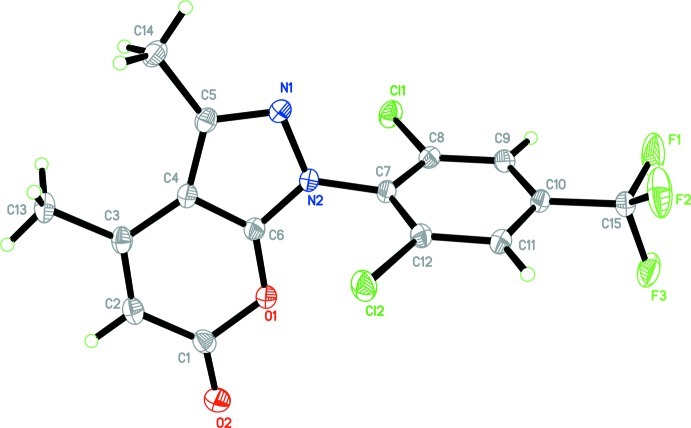
The molecular structure of the title compound, showing 50% probability displacement ellipsoids.

**Fig. 2.**
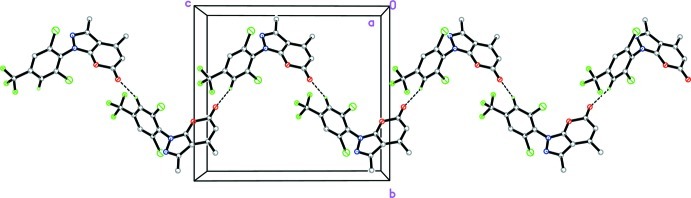
The crystal packing of the title compound, viewed along the a axis, showing the chain along the c axis. H atoms not involved in the intermolecular interactions (dashed lines) have been omitted for clarity.

## supplementary crystallographic information

### Comment

Pyrazolone moiety is a pyrazole derivative, containing a five-membered lactam ring with two nitrogen atoms and a ketone group in the same molecule. Edaravone, 3-methyl-1-phenyl-2-pyrazolin-5-one, is a strong novel free radical scavenger and was used for treatment of patients with acute brain infarction (Kokura *et al.*, 2005). The radical scavenging capacity and molecular binding activities of various derivatives of pyrazol-5-ols were reported (Sarojini *et al.*, 2010). In an attempt to synthesize pyrazolone derivative, the title compound was formed when ethyl acetoacetate was taken in 1: 2 molar ratio. Similar product formation was reported by Vaid *et al.* (1986). The crystal structure of 3,4-dimethyl-1-(2-pyridyl)pyrano[2,3-*c*]pyrazol-6(1*H*)-one was reported by Ramsay & Steel (1985) and the structure of 3,4-dimethyl-1-phenylpyrano[2,3-*c*]-pyrazol-6(1*H*)-one was reported by Ahmad *et al.* (2011). The title compound 1-(2,6-dichloro-4-(trifluoromethyl)phenyl)-3,4- dimethylpyrano[2,3-*c*]pyrazol-6(1*H*)-one [C_15_H_9_Cl_2_F_3_N_2_O_2_], was prepared by the reaction of 2,6-dichloro-4-trifluoromethyl-phenyl hydrazine with excess of ethyl acetoacetate.

In the title compound, Fig. 1, the 1,6-dihydropyrano[2,3-*c*]pyrazole ring system (C1–C5/N1/N2/C6/O1) is almost planar with the maximum deviation of 0.0226 (14) Å at atom C2 and forms a dihedral angle of 69.90 (6)° with the benzene ring (C7–C12). Bond lengths (Allen *et al.*, 1987) and angles are within the normal ranges.

In the crystal packing as shown in Fig. 2, the molecules are linked into chains along the *c* axis by the intermolecular C11—H11A···O2 hydrogen bonds (Table 1).

### Experimental

(2,6-Dichloro-4-trifluoromethyl)phenyl hydrazine (1.225 g, 0.005 mol) was added to ethyl acetoacetate (1.3 g, 0.01 mol) and charged in a microwave for 3 minutes at 360 W. The reaction mixture was quenched into ether and kept for some time to get the residue. The residue was recrystallized by slow evaporation from ethanol to give block-shaped orange crystals suitable for X-ray diffraction. *M.p.* = 431 K.

### Refinement

All H atoms were positioned geometrically and were refined with a riding model with *U*_iso_(H) = 1.2 or 1.5 *U*_eq_(C) (C—H = 0.95 or 0.98 Å). A rotating group model was applied to the methyl groups.

### Crystal data

**Table:**

C_15_H_9_Cl_2_F_3_N_2_O_2_	*F*(000) = 1520
*M**_r_* = 377.14	*D*_x_ = 1.662 Mg m^−^^3^
Orthorhombic, *P**b**c**a*	Mo *K*α radiation, λ = 0.71073 Å
Hall symbol: -P 2ac 2ab	Cell parameters from 9873 reflections
*a* = 13.3348 (2) Å	θ = 2.5–32.6°
*b* = 14.2045 (2) Å	µ = 0.48 mm^−^^1^
*c* = 15.9132 (3) Å	*T* = 100 K
*V* = 3014.19 (8) Å^3^	Block, orange
*Z* = 8	0.44 × 0.31 × 0.26 mm

### Data collection

**Table:**

Bruker SMART APEXII CCD area-detector diffractometer	4382 independent reflections
Radiation source: fine-focus sealed tube	3706 reflections with *I* > 2σ(*I*)
Graphite monochromator	*R*_int_ = 0.030
φ and ω scans	θ_max_ = 30.0°, θ_min_ = 2.5°
Absorption correction: multi-scan (*SADABS*; Bruker, 2009)	*h* = −18→18
*T*_min_ = 0.819, *T*_max_ = 0.888	*k* = −19→19
24894 measured reflections	*l* = −22→20

### Refinement

**Table:**

Refinement on *F*^2^	Primary atom site location: structure-invariant direct methods
Least-squares matrix: full	Secondary atom site location: difference Fourier map
*R*[*F*^2^ > 2σ(*F*^2^)] = 0.036	Hydrogen site location: inferred from neighbouring sites
*wR*(*F*^2^) = 0.096	H-atom parameters constrained
*S* = 1.03	*w* = 1/[σ^2^(*F*_o_^2^) + (0.0445*P*)^2^ + 2.1563*P*] where *P* = (*F*_o_^2^ + 2*F*_c_^2^)/3
4382 reflections	(Δ/σ)_max_ = 0.001
219 parameters	Δρ_max_ = 0.43 e Å^−^^3^
0 restraints	Δρ_min_ = −0.52 e Å^−^^3^

### Special details

**Table:**

Experimental. The crystal was placed in the cold stream of an Oxford Cryosystems Cobra open-flow nitrogen cryostat (Cosier & Glazer, 1986) operating at 100.0 (1) K.
Geometry. All e.s.d.'s (except the e.s.d. in the dihedral angle between two l.s. planes) are estimated using the full covariance matrix. The cell e.s.d.'s are taken into account individually in the estimation of e.s.d.'s in distances, angles and torsion angles; correlations between e.s.d.'s in cell parameters are only used when they are defined by crystal symmetry. An approximate (isotropic) treatment of cell e.s.d.'s is used for estimating e.s.d.'s involving l.s. planes.
Refinement. Refinement of *F*^2^ against ALL reflections. The weighted *R*-factor *wR* and goodness of fit *S* are based on *F*^2^, conventional *R*-factors *R* are based on *F*, with *F* set to zero for negative *F*^2^. The threshold expression of *F*^2^ > σ(*F*^2^) is used only for calculating *R*-factors(gt) *etc*. and is not relevant to the choice of reflections for refinement. *R*-factors based on *F*^2^ are statistically about twice as large as those based on *F*, and *R*- factors based on ALL data will be even larger.

### Fractional atomic coordinates and isotropic or equivalent isotropic displacement parameters (Å^2^)

**Table:**

	*x*	*y*	*z*	*U*_iso_*/*U*_eq_	
Cl1	0.05786 (3)	0.14096 (3)	0.71268 (2)	0.02478 (10)	
Cl2	0.32815 (3)	0.42169 (3)	0.68567 (2)	0.02497 (10)	
F1	0.01024 (12)	0.35846 (9)	0.97088 (8)	0.0559 (4)	
F2	0.14301 (8)	0.44227 (10)	0.97926 (7)	0.0426 (3)	
F3	0.01721 (9)	0.49137 (8)	0.91002 (6)	0.0399 (3)	
O1	0.20606 (8)	0.33302 (7)	0.52607 (6)	0.0197 (2)	
O2	0.18623 (9)	0.41836 (8)	0.41029 (7)	0.0260 (2)	
N1	0.29995 (9)	0.15794 (8)	0.66209 (8)	0.0187 (2)	
N2	0.23949 (9)	0.23515 (8)	0.64239 (7)	0.0181 (2)	
C1	0.23104 (11)	0.35320 (10)	0.44100 (9)	0.0202 (3)	
C2	0.30521 (11)	0.29344 (10)	0.40143 (9)	0.0209 (3)	
H2A	0.3237	0.3070	0.3451	0.025*	
C3	0.35023 (11)	0.21887 (10)	0.43997 (9)	0.0189 (3)	
C4	0.32303 (10)	0.20139 (10)	0.52589 (9)	0.0166 (3)	
C5	0.34852 (11)	0.13725 (10)	0.59193 (9)	0.0180 (3)	
C6	0.25427 (11)	0.26101 (9)	0.56213 (9)	0.0174 (3)	
C7	0.19107 (10)	0.28382 (9)	0.70869 (9)	0.0167 (3)	
C8	0.10967 (10)	0.24338 (9)	0.75021 (9)	0.0173 (2)	
C9	0.06951 (11)	0.28556 (10)	0.82140 (9)	0.0184 (3)	
H9A	0.0149	0.2574	0.8503	0.022*	
C10	0.11052 (11)	0.36948 (10)	0.84946 (9)	0.0176 (3)	
C11	0.18835 (11)	0.41405 (10)	0.80713 (9)	0.0189 (3)	
H11A	0.2135	0.4730	0.8258	0.023*	
C12	0.22825 (10)	0.37016 (10)	0.73692 (9)	0.0179 (3)	
C13	0.42522 (12)	0.15731 (12)	0.39669 (10)	0.0247 (3)	
H13A	0.4314	0.1766	0.3378	0.037*	
H13B	0.4029	0.0916	0.3994	0.037*	
H13C	0.4905	0.1634	0.4245	0.037*	
C14	0.41785 (12)	0.05479 (11)	0.59007 (10)	0.0227 (3)	
H14A	0.4190	0.0247	0.6455	0.034*	
H14B	0.4856	0.0761	0.5755	0.034*	
H14C	0.3946	0.0094	0.5480	0.034*	
C15	0.07067 (11)	0.41500 (10)	0.92787 (9)	0.0199 (3)	

### Atomic displacement parameters (Å^2^)

**Table:**

	*U*^11^	*U*^22^	*U*^33^	*U*^12^	*U*^13^	*U*^23^
Cl1	0.02666 (18)	0.01914 (17)	0.02854 (19)	−0.00419 (13)	0.00424 (15)	−0.00636 (13)
Cl2	0.02532 (18)	0.02462 (18)	0.02499 (19)	−0.00624 (13)	0.00913 (14)	−0.00204 (13)
F1	0.0876 (10)	0.0374 (6)	0.0427 (7)	−0.0230 (6)	0.0449 (7)	−0.0153 (5)
F2	0.0261 (5)	0.0781 (9)	0.0235 (5)	0.0124 (5)	−0.0059 (4)	−0.0203 (5)
F3	0.0531 (7)	0.0427 (6)	0.0240 (5)	0.0310 (5)	−0.0038 (5)	−0.0093 (4)
O1	0.0229 (5)	0.0196 (5)	0.0166 (5)	0.0040 (4)	0.0025 (4)	0.0000 (4)
O2	0.0315 (6)	0.0244 (5)	0.0221 (5)	0.0033 (4)	0.0003 (5)	0.0033 (4)
N1	0.0205 (6)	0.0167 (5)	0.0190 (6)	0.0034 (4)	0.0012 (5)	−0.0010 (4)
N2	0.0219 (6)	0.0168 (5)	0.0156 (5)	0.0033 (4)	0.0039 (5)	−0.0012 (4)
C1	0.0234 (7)	0.0205 (6)	0.0169 (6)	−0.0023 (5)	0.0004 (5)	0.0000 (5)
C2	0.0216 (7)	0.0246 (7)	0.0166 (6)	−0.0021 (5)	0.0025 (5)	−0.0023 (5)
C3	0.0173 (6)	0.0214 (6)	0.0180 (6)	−0.0031 (5)	0.0024 (5)	−0.0052 (5)
C4	0.0160 (6)	0.0171 (6)	0.0168 (6)	−0.0003 (5)	0.0019 (5)	−0.0028 (5)
C5	0.0174 (6)	0.0169 (6)	0.0196 (7)	−0.0006 (5)	0.0001 (5)	−0.0037 (5)
C6	0.0193 (6)	0.0162 (6)	0.0167 (6)	0.0000 (5)	0.0014 (5)	−0.0017 (5)
C7	0.0182 (6)	0.0171 (6)	0.0147 (6)	0.0033 (5)	0.0013 (5)	−0.0016 (5)
C8	0.0181 (6)	0.0158 (6)	0.0179 (6)	0.0003 (5)	−0.0001 (5)	−0.0013 (5)
C9	0.0181 (6)	0.0190 (6)	0.0182 (6)	0.0014 (5)	0.0029 (5)	0.0007 (5)
C10	0.0195 (6)	0.0180 (6)	0.0152 (6)	0.0050 (5)	0.0022 (5)	−0.0014 (5)
C11	0.0218 (6)	0.0167 (6)	0.0182 (6)	0.0010 (5)	0.0018 (5)	−0.0027 (5)
C12	0.0183 (6)	0.0181 (6)	0.0174 (6)	0.0001 (5)	0.0038 (5)	0.0001 (5)
C13	0.0230 (7)	0.0294 (8)	0.0216 (7)	0.0033 (6)	0.0062 (6)	−0.0045 (6)
C14	0.0231 (7)	0.0208 (7)	0.0241 (7)	0.0059 (5)	0.0004 (6)	−0.0032 (5)
C15	0.0229 (7)	0.0212 (6)	0.0157 (6)	0.0027 (5)	0.0025 (5)	−0.0020 (5)

### Geometric parameters (Å, º)

**Table:**

Cl1—C8	1.7176 (14)	C4—C5	1.432 (2)
Cl2—C12	1.7249 (14)	C5—C14	1.4926 (19)
F1—C15	1.3277 (18)	C7—C8	1.3946 (19)
F2—C15	1.3227 (18)	C7—C12	1.3971 (19)
F3—C15	1.3288 (17)	C8—C9	1.3890 (19)
O1—C6	1.3375 (17)	C9—C10	1.386 (2)
O1—C1	1.4233 (17)	C9—H9A	0.9500
O2—C1	1.2053 (18)	C10—C11	1.390 (2)
N1—C5	1.3238 (18)	C10—C15	1.5023 (19)
N1—N2	1.3967 (16)	C11—C12	1.3858 (19)
N2—C6	1.3436 (18)	C11—H11A	0.9500
N2—C7	1.4170 (17)	C13—H13A	0.9800
C1—C2	1.448 (2)	C13—H13B	0.9800
C2—C3	1.363 (2)	C13—H13C	0.9800
C2—H2A	0.9500	C14—H14A	0.9800
C3—C4	1.436 (2)	C14—H14B	0.9800
C3—C13	1.496 (2)	C14—H14C	0.9800
C4—C6	1.3749 (18)		
			
C6—O1—C1	116.71 (11)	C10—C9—H9A	120.6
C5—N1—N2	105.51 (12)	C8—C9—H9A	120.6
C6—N2—N1	110.08 (11)	C9—C10—C11	122.04 (13)
C6—N2—C7	129.89 (12)	C9—C10—C15	119.89 (13)
N1—N2—C7	118.62 (11)	C11—C10—C15	118.07 (13)
O2—C1—O1	115.10 (13)	C12—C11—C10	118.21 (13)
O2—C1—C2	127.79 (14)	C12—C11—H11A	120.9
O1—C1—C2	117.11 (12)	C10—C11—H11A	120.9
C3—C2—C1	124.11 (13)	C11—C12—C7	121.19 (13)
C3—C2—H2A	117.9	C11—C12—Cl2	119.14 (11)
C1—C2—H2A	117.9	C7—C12—Cl2	119.64 (11)
C2—C3—C4	116.84 (13)	C3—C13—H13A	109.5
C2—C3—C13	122.77 (14)	C3—C13—H13B	109.5
C4—C3—C13	120.39 (13)	H13A—C13—H13B	109.5
C6—C4—C5	104.03 (12)	C3—C13—H13C	109.5
C6—C4—C3	117.46 (13)	H13A—C13—H13C	109.5
C5—C4—C3	138.49 (13)	H13B—C13—H13C	109.5
N1—C5—C4	111.20 (12)	C5—C14—H14A	109.5
N1—C5—C14	119.61 (13)	C5—C14—H14B	109.5
C4—C5—C14	129.18 (13)	H14A—C14—H14B	109.5
O1—C6—N2	123.12 (12)	C5—C14—H14C	109.5
O1—C6—C4	127.72 (13)	H14A—C14—H14C	109.5
N2—C6—C4	109.15 (12)	H14B—C14—H14C	109.5
C8—C7—C12	119.05 (12)	F2—C15—F1	107.52 (13)
C8—C7—N2	120.43 (12)	F2—C15—F3	106.52 (13)
C12—C7—N2	120.40 (12)	F1—C15—F3	106.16 (13)
C9—C8—C7	120.59 (13)	F2—C15—C10	112.43 (12)
C9—C8—Cl1	119.59 (11)	F1—C15—C10	112.49 (12)
C7—C8—Cl1	119.82 (11)	F3—C15—C10	111.32 (12)
C10—C9—C8	118.81 (13)		
			
C5—N1—N2—C6	−1.53 (16)	C3—C4—C6—N2	−178.89 (12)
C5—N1—N2—C7	−169.29 (12)	C6—N2—C7—C8	122.18 (17)
C6—O1—C1—O2	179.89 (13)	N1—N2—C7—C8	−72.87 (17)
C6—O1—C1—C2	0.64 (18)	C6—N2—C7—C12	−62.0 (2)
O2—C1—C2—C3	−177.74 (15)	N1—N2—C7—C12	102.97 (16)
O1—C1—C2—C3	1.4 (2)	C12—C7—C8—C9	−3.5 (2)
C1—C2—C3—C4	−1.7 (2)	N2—C7—C8—C9	172.37 (13)
C1—C2—C3—C13	178.78 (14)	C12—C7—C8—Cl1	175.99 (11)
C2—C3—C4—C6	−0.07 (19)	N2—C7—C8—Cl1	−8.11 (19)
C13—C3—C4—C6	179.51 (13)	C7—C8—C9—C10	1.3 (2)
C2—C3—C4—C5	−178.26 (16)	Cl1—C8—C9—C10	−178.27 (11)
C13—C3—C4—C5	1.3 (3)	C8—C9—C10—C11	2.0 (2)
N2—N1—C5—C4	1.45 (16)	C8—C9—C10—C15	−178.05 (13)
N2—N1—C5—C14	−177.79 (12)	C9—C10—C11—C12	−2.9 (2)
C6—C4—C5—N1	−0.87 (16)	C15—C10—C11—C12	177.19 (13)
C3—C4—C5—N1	177.48 (16)	C10—C11—C12—C7	0.5 (2)
C6—C4—C5—C14	178.29 (14)	C10—C11—C12—Cl2	−177.47 (11)
C3—C4—C5—C14	−3.4 (3)	C8—C7—C12—C11	2.6 (2)
C1—O1—C6—N2	178.79 (13)	N2—C7—C12—C11	−173.26 (13)
C1—O1—C6—C4	−2.6 (2)	C8—C7—C12—Cl2	−179.39 (11)
N1—N2—C6—O1	179.89 (12)	N2—C7—C12—Cl2	4.71 (19)
C7—N2—C6—O1	−14.2 (2)	C9—C10—C15—F2	133.24 (15)
N1—N2—C6—C4	1.02 (16)	C11—C10—C15—F2	−46.84 (18)
C7—N2—C6—C4	166.98 (14)	C9—C10—C15—F1	11.7 (2)
C5—C4—C6—O1	−178.93 (14)	C11—C10—C15—F1	−168.40 (14)
C3—C4—C6—O1	2.3 (2)	C9—C10—C15—F3	−107.33 (16)
C5—C4—C6—N2	−0.13 (15)	C11—C10—C15—F3	72.59 (17)

### Hydrogen-bond geometry (Å, º)

**Table:**

*D*—H···*A*	*D*—H	H···*A*	*D*···*A*	*D*—H···*A*
C11—H11*A*···O2^i^	0.95	2.44	3.3405 (18)	157

Symmetry code: (i) −*x*+1/2, −*y*+1, *z*+1/2.
